# Evaluating an internal quality assurance process for achieving national accreditation standards in midwifery education: a study protocol

**DOI:** 10.1080/16549716.2025.2463234

**Published:** 2025-02-13

**Authors:** Frida Berg, Kerstin Erlandsson, Paridhi Jha, Helena Wigert, Bharati Sharma, Malin Bogren

**Affiliations:** a Institute of Health and Care Sciences, Sahlgrenska Academy, University of Gothenburg, Gothenburg, Sweden; b School of Health and Welfare, Dalarna University, Falun, Sweden; c Foundation for Research in Health Systems, Indian Society for Health Administrators, Bangalore, India; d Indian Institute of Public Health, Gandhinagar, Gujarat, India

**Keywords:** Accreditation, implementation science, internal quality assurance, midwifery education, process evaluation

## Abstract

The World Health Organization and the International Confederation of Midwives emphasize the importance of accreditation to enhance quality in midwifery education. In midwifery education programmes, internal self-assessments are used to meet accreditation criteria. However, research on this topic is scarce. Therefore, this paper describes how we plan to conduct an evaluation of an internal quality assurance process in midwifery education aimed at achieving national accreditation standards in Bangladesh. This study has a longitudinal exploratory design and will be guided by the principles of process evaluation of complex interventions. An internal quality assurance self-assessment intervention will be introduced at 31 private and public education institutions in Bangladesh. To ensure a sustainable implementation, the Plan-Do-Study-Act cycle will be introduced. Data will be collected using self-administered questionnaires and focus group discussions with midwifery faculty and final-semester students. Descriptive statistics and regression models will be performed for the quantitative data, and the qualitative data will be analysed using content analysis. It is anticipated that, without internal quality assurance of midwifery education programmes, accreditation alone is unlikely to enhance quality. We aspire for this research project to illustrate a process that the midwifery institutes can implement themselves for sustainable transformation towards high-quality midwifery education in countries where such internal quality assurance processes have not yet been integrated into the education system.

**Trial registration**: The study was registered retrospectively with the ISRCTN registry on 26 August 2024. The registration number is: ISRCTN14492910.

## Paper context

**Main findings**: We aspire for this research project to illustrate a process for sustainable transformation towards high-quality midwifery education.

**Added knowledge**: The use of implementation science is a new approach in midwifery education that explores the implementation of a quality assurance process and its mechanism of impact and outcomes.

**Global health impact for policy and action**: Our research findings can serve as a model to inform policy-makers advancing towards quality midwifery education.

## Background

In midwifery education programmes in several high-income countries, recurrent self-assessments are conducted to ensure a learning environment in which the content of programmes, learning opportunities, and facilities effectively meet education goals [1]. These self-assessments offer valuable feedback and suggestions for enhancing quality in education programmes in preparation for the establishment of formal accreditation [1–3]. Both the World Health Organization and the International Confederation of Midwives emphasize the critical role of accreditation in improving the quality of midwifery education [4–6]. Despite this emphasis, there is a notable gap in research on the effectiveness of self-assessments for continuous quality improvement in midwifery education, particularly before and after formal accreditation. This highlights the need to scientifically evaluate internal quality assurance self-assessments for achieving national accreditation standards in midwifery education programmes. In our study, Bangladesh is used as an example.

The International Confederation of Midwives has established education standards to strengthen midwifery globally by promoting quality education programmes that prepare midwives in line with the International Confederation of Midwives definition of a midwife [6]. These standards consist of six categories: i) Programme governance, ii) Faculty, iii) Students, iv) Midwifery programme and curriculum, v) Resources, and vi) Quality improvements. These standards are grounded in the best available evidence and serve as a benchmark for minimal achievement in midwifery education. The standards offer a structured approach for developing, implementing, and assessing programme quality while ensuring alignment with regulatory requirements [6]. By adhering to these standards, midwifery education programmes can demonstrate accountability to the public and adapt to national and local workforce demands. In essence, these standards encompass criteria such as programme duration, entry-level skills, core competencies, educator qualifications, and ongoing competency maintenance for fully qualified midwives. The International Confederation of Midwives global standards for midwifery education [6] can be used to develop an internal quality education assurance process, such as a self-assessment at an education institution, while at the same time taking the national context into consideration.

Less than half of the world’s countries have an accreditation system for midwifery education, and it is evident that midwifery education programmes, particularly in low-income countries, do not fully meet accreditation standards. Accreditation formally acknowledges procedures aimed at ensuring that education programmes adhere to national established standards, thereby enabling graduates to attain the agreed-upon minimum level of competence [7,8]. Research on accreditation’s impact on education quality exists [8–11], but there is a lack of studies on continuous improvement such as self-assessments in midwifery education before and after accreditation.

Investment in midwifery education by the government of Bangladesh over the past decade [12] has been aimed at improving maternal and newborn mortality and morbidity, yielding positive results [9,13–18]. However, progress has stagnated [19]. To meet the Agenda 2030 target of reducing the maternal mortality ratio to less than 70 deaths per 100,000 live births, the government has initiated measures for an internal quality assurance self-assessment process for midwifery education programmes, in addition to the national accreditation system. This paper describes how we plan to conduct an evaluation of the internal quality assurance process in midwifery education aimed at achieving national accreditation standards in Bangladesh. The specific aims are to:

Evaluate to what extent education standards are met in the internal quality assurance process at midwifery education institutions.

Determine the effectiveness of the internal quality assurance process in producing competent and confident educators and midwives.

Identify contextual factors influencing the internal quality assurance process to reach national accreditation standards for midwifery education.

The term ‘quality assurance’ is used in this paper to describe all activities within the recurrent improvement cycles.

### Hypothesis

We hypothesize that implementing an internal quality assurance process will improve the quality of midwifery education programmes, producing competent and confident educators and midwives and thereby meeting national accreditation standards.

## Methods

### Research design

**Figure 1.**
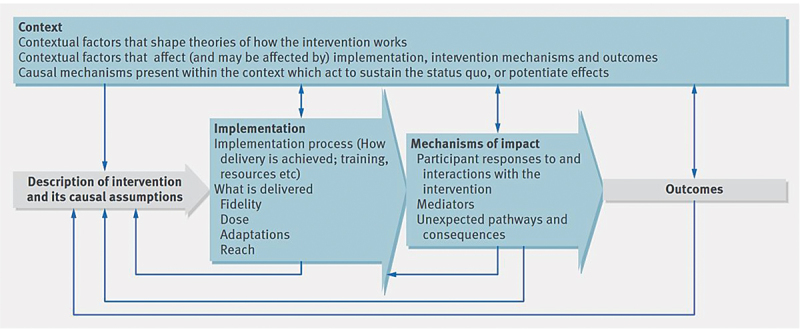
Key functions of process evaluation and relations among them as described by Moore et al. [20].

The evaluation of the internal quality assurance process for midwifery education will use a longitudinal exploratory design guided by the principles of process evaluations of complex interventions [20]. This includes a description of an intervention and contextual factors that may affect the implementation process of the intervention, what is delivered (fidelity, dose, adaptations, and reach), the mechanisms of the impact that influence the intervention activities (responses, mediators, unexpected pathways, and consequences), and the outcomes of the intervention [20]. For details, see Figure 1.

#### Setting

Midwifery education in Bangladesh is currently delivered at 147 institutes (60 public, 87 private) across the country, consisting of 60% clinical practice and 40% theory leading to a diploma degree after three years of study. Each of these programmes admits 25 to 50 students annually, attracting students from rural and urban areas. The educators hold a PhD or master’s degree, either in Sexual Reproductive and Perinatal Health, Hospital Management, Women’s Health, Nursing, or Public Health [11,14,21]. The internal quality assurance process will be initiated in 31 of the 147 public and private education institutions selected by the government of Bangladesh. The United Nations Population Fund in Bangladesh and the Swedish Development Cooperation Agency support these institutions in strengthening midwifery education and services in the districts where they are located.

#### The internal quality assurance process intervention for midwifery education

The intervention consists of an internal quality assurance process to be integrated in education institutions to enhance the quality of midwifery education. Through a recurrent self-assessment, the faculty assess their respective education programmes against national and international standards [22]. The faculty within the midwifery education programmes internally analyse the achievements of the self-assessment, indicating which standards are met, partially met, and unmet and what changes need to be prioritized in the next cycle. Based on the yearly self-assessment findings, the faculty make a recurrent activity plan. The learning from one assessment guides the plan that follows [23].

The implementation strategies include the following steps:

Update an existing tool to become a quality assurance assessment tool. The updated tool will be aligned with the new International Confederation of Midwives education standards [6] and the Bangladeshi accreditation standards for midwifery education programmes [9,11,17].

Train quality assurance task force committee members, consisting of midwifery educators (*n* = 21) selected by the government, representing both public and private education institutions. They will be trained on the quality assurance assessment tool during a four-day workshop. Upon completion, they will collect baseline data from all 31 sites.

Implement the Plan-Do-Study-Act (PDSA) cycle at all 31 institutions. The PDSA cycle is a four-step approach commonly used for quality improvement [23]. Through this approach, education institutions can ensure that their midwifery programmes meet education standards, fostering an environment of continuous improvement in midwifery education. The following steps will be applied:

Initially, the faculty members, such as educators, clinical supervisors, midwifery students, and library, computer lab, and clinical simulation managers, will conduct a yearly self-assessment of their education programmes against education standards. Following the self-assessment, an internal analysis of the findings will identify which areas require prioritization for improvement. This will provide a clear understanding of the changes needed to meet the standards.

In the Plan phase, based on the self-assessment, the faculty will develop an activity plan. This plan will address the prioritized areas for improvement, outlining specific actions and strategies to be implemented over the coming year to reach the standards.

In the Do phase, the faculty will execute the activity plan and conduct the next assessment about nine months later.

In the Study phase, the collected data will be analysed after the subsequent assessment has been conducted. The results will show whether the previously set standards were met, whether the recommended improvements were adopted, and whether any remaining needs were identified.

In the Act phase, based on the self-assessment findings, the activity plan will be reviewed. Insights gained from each cycle of the assessment will guide the subsequent cycle. In this process, the activities will be adopted, adapted, or abandoned. The continuous internal feedback loops will ensure that midwifery education is aligned with education standards [23].

**Figure 2.**
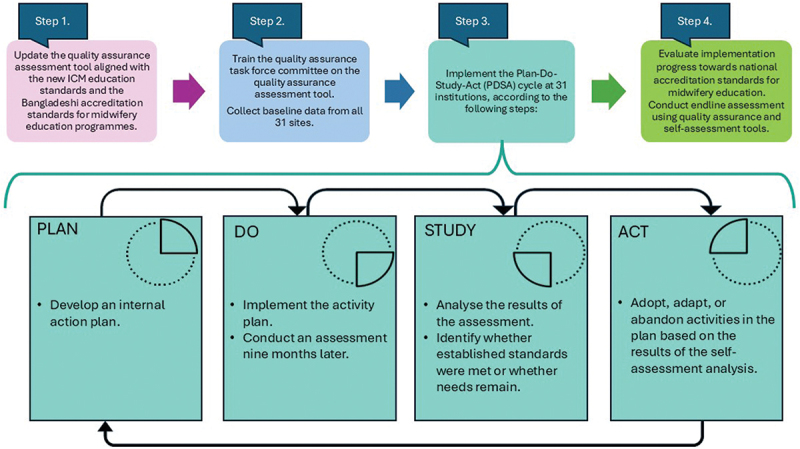
Overview of the implementation strategies.

Conduct an endline assessment at each institution using the quality assurance assessment tool and the self-assessment tool for midwifery educators and final-semester students. This evaluates the implementation process and the progress made in reaching the national accreditation standards in midwifery education (Figure 2).

### Data collection

Both quantitative components, using three self-assessment tools, and qualitative data, using focus group discussions (FGD), will be used [20].

#### Quantitative data

To measure the extent to which education standards are being met in the internal quality assurance process at the education institutions, a validated internal quality assurance assessment tool will be used [22]. The tool consists of 56 statements across six categories, built on International Confederation of Midwives Global Standards for midwifery education: i) Programme governance; ii) Faculty; iii) Students; iv) Midwifery programme and curriculum; v) Resources; and vi) Quality improvements [22], including scoring alternatives ‘Meeting the standard’, ‘Partially meeting the standard’, and ‘Unmet standard’. A team at each site, selected by the principal, will collaboratively complete the tool to ensure a comprehensive assessment of the current situation at each institution. The team may consist of educators, clinical supervisors, librarians, persons responsible for the computer lab and the clinical simulation lab, and midwifery students.

To determine the effectiveness of the internal quality assurance process in producing competent and confident midwifery educators, a digital self-assessment tool that has been contextualized and validated for Bangladesh in English and Bangla will be used. This tool records midwifery educators’ self-assessed confidence and competence in delivering the World Health Organization’s educator competencies [24,25]. The tool consists of 97 items distributed across nine core competencies and includes socio-demographic and work-related questions. The government will distribute the digital self-assessment tool to all midwife educators (*n* = 170–230) at the selected institutes following their consent to participate.

To evaluate the effectiveness of the internal quality assurance process in producing competent and confident midwives, a digital self-assessment tool in Bangla will be developed. The existing tool is based on the 2014 International Confederation of Midwives Essential Competencies [26] and is validated by the World Health Organization South-East Asia Region [25] for professional midwives, and the contextual items will be modified for use among midwifery students in Bangladesh. It will include 230 items across the seven core competencies, including a seven-point Likert scale for items, a socio-demographic profile section, and some contextual questions. This tool will be administered by the quality assurance task force committee to all final-semester midwifery students (*n* = 775–1550) following their consent. The latter two tools will be distributed at each selected institution at two time points: at the start of the study (baseline) and at the end of the study (endline).

#### Qualitative data

Focus group discussions (FGD) will be conducted to identify contextual factors that influence the internal quality assurance process and the mechanism of impact influencing the implementation of the intervention. Ten FGDs will be conducted comprising six to eight participants representing two education institutions that scored themselves at a higher level using the quality assurance self-assessment tool [22], three institutions that scored themselves at a medium level, and five that scored themselves at a lower level. In addition, FGDs (*n* = 4) will be conducted with the quality assurance task force committee members.

An interview guide will be developed guided by the principles of process evaluations of complex interventions [20], and one interview guide will be designed to cover the International Confederation of Midwives Global Standard for midwifery education [6]. All FGDs will be conducted in English and will be audio recorded.

### Data analysis

Quantitative data will be entered into IBM’s Statistical Package for Social Science (SPSS) Statistics (version 25) for descriptive statistics (frequencies, percentages, mean, and standard deviation). Overall scores of the quality assurance assessment tool will be categorized as low-standard (<60%), medium-standard (60% - <80%), or high-standard (≥80%). Low scores indicate significant resources needed, medium scores suggest moderate resources, and high scores imply fewer resources needed to meet Bangladesh’s national accreditation standards [9,17,22].

For the self-assessment tools for midwifery educators and students, the self-assessed confidence and competence scores will be calculated for each core competency. Principal component analysis will be used for data reduction, while frequencies, percentages, means, and standard deviations will be calculated to describe the socio-demographic and/or work profiles of the participants [27]. Non-parametric chi square tests will be performed to explore associations between the socio-demographic variables and the confidence and competence scores. Correlations between confidence and competence scores will also be explored. Based on the results, regression models will be used.

Qualitative data analysis will follow the principles of content analysis, based on Elo and Kyngäs [28]. The process involves reading through the entire text multiple times to understand it in its entirety, identifying meaning units answering the aim, and thereafter, coding and categorization.

## Discussion

Introducing an internal quality assurance process in Bangladesh can potentially transform midwifery education sustainably towards higher quality [22,29]. In Bangladesh, the Ministry of Health, the Bangladesh Nursing and Midwifery Council, and the Directorate General of Nursing and Midwifery (DGNM) are responsible for ensuring the quality of midwifery education. Our research project supports these entities with scientific data and new knowledge which will be used for disseminating the internal quality assurance process across all midwifery education institutions in Bangladesh [22]. It is expected that this new knowledge will contribute to the common understanding of quality assurance for learning and teaching across the institutions.

While the Bangladeshi government has initiated the national accreditation system, they have at the same time initiated an internal quality assurance process in midwifery education. Introducing the internal quality assurance process in parallel with the accreditation allows the Bangladeshi education system to demonstrate quality and increase transparency, thus supporting the building of mutual trust and better recognition of the midwifery programmes. In contrast, introducing accreditation alone could result in the closure of education programmes that may not meet the accreditation standards [11], leaving students without education and the country without enough midwives. Therefore, it can be argued that an internal quality assurance process must be implemented in parallel with continuous improvement cycles to meet national accreditation standards before the national accreditation reviews.

Inspired by the European Association for Quality Assurance in Higher Education [1], we will develop an internal quality assurance process intervention through dialogue with representatives from universities and the Ministry of Health in Bangladesh, forming a quality assurance task force committee. One context does not guarantee effectiveness elsewhere without appropriate adaptation [20]. Therefore, the internal quality assurance process intervention, its implementation strategies, and the tools to be used, are being developed in collaboration with the quality assurance task force committee and are thereby being tailored to meet the specific context of Bangladesh. One implementation strategy is the use of the PDSA cycle for continuous improvement [23]. By using PDSA cycles, education institutions can systematically monitor and evaluate their activities, using the results to achieve high-quality education and effective practice changes. With this approach, a contextualized tool will be used in the internal quality assurance self-assessments. This tool helps institutions identify strengths and weaknesses effectively, enabling them to independently prioritize and implement action plans [22].

Despite anecdotal benefits of internal quality assurance self-assessments in transforming midwifery education towards higher quality in both high- and low-income countries, there has been little research in this area, and scientific evidence is lacking. This lack of prior research constitutes the main novelty of this project. Using an implementation science framework [20] in an internal quality assurance process initiative in a low-income country is a new approach that scientifically explores the implementation of a quality assurance process and its mechanisms of impact and outcomes. These findings can provide lessons learned and guide other education institutions and governments that want to implement a similar process. This is noteworthy in that many countries in South-East Asia are currently transitioning to a midwifery model of care and will potentially benefit from this work.

### Impact of the research

#### At the policy level

Accreditation aims to ensure that education programmes achieve a minimum level of competence. Our research findings can demonstrate the usefulness of establishing an internal quality assurance process for sustainable transformation towards high-quality midwifery education in countries where such a process has not yet been integrated into the education system. This study could further provide guidance for policy- and decision-makers in designing a quality assurance process at the institutional level in collaboration with midwifery faculty.

#### At the institutional level

We aspire for this study to serve as a model demonstrating an internal quality assurance process within an existing accreditation system to enhance the quality of midwifery education. It is anticipated that implementing this internal quality assurance process in Bangladesh’s existing education system can ensure that education institutions across the country adhere to education standards [30].

#### At the society level

The quality of midwifery education has a profound impact on maternal and newborn healthcare outcomes in the society [30,31]. Through this quality assurance process intervention, it is expected that the challenges related to the quality of the midwifery education in Bangladesh, including the South-East Asia region, can contribute to midwives’ competence and confidence and thereby effectively carry out the midwives’ role within maternal and newborn health care [32–35].
